# Changes in Levels of Nerve Growth Factor in Nasal Secretions after Capsaicin Inhalation in Patients with Airway Symptoms from Scents and Chemicals

**DOI:** 10.1289/ehp.7657

**Published:** 2005-03-17

**Authors:** Eva Millqvist, Ewa Ternesten-Hasséus, Arne Ståhl, Mats Bende

**Affiliations:** ^1^Asthma and Allergy Research Group, Department of Respiratory Medicine and Allergy, ahlgrenska University Hospital, Göteborg, Sweden; ^2^Allergy Centre, Central Hospital, Skövde, Sweden

**Keywords:** airway symptoms, chemical intolerance, multiple chemical sensitivity, nasal lavage fluid, nerve growth factor, sensory hyperreactivity

## Abstract

Patients complaining of upper and lower airway symptoms caused by scents and chemicals have previously been shown to have increased cough sensitivity to inhaled capsaicin, but the precise mechanisms behind this reaction are unknown. Hypothesizing that a neurochemical alteration related to sensory hyperreactivity (SHR) of the airway mucosa occurs, we measured levels of nerve growth factor (NGF) in nasal lavage fluid (NAL) before and after capsaicin inhalation provocations and related the capsaicin cough sensitivity to the NGF levels. Thirteen patients with SHR and 14 control subjects were provoked with capsaicin inhalation at three different doses. We measured NGF in NAL before and after provocation and recorded cough and capsaicin-induced symptoms. All subjects demonstrated a dose-dependent cough response to capsaicin inhalation, with a more pronounced effect in patients than in controls. Basal levels of NGF were significantly lower in the patient group than in the control subjects (*p* < 0.01). After capsaicin provocation, the patients showed a significant increase in NGF (*p* < 0.01), which was related to capsaicin cough sensitivity. The findings demonstrate that, in patients with airway symptoms induced by scents and chemicals, SHR is real and measurable, demonstrating a pathophysiology in the airways of these patients compared to healthy subjects.

According to several epidemiologic studies, chemical intolerance is a common problem (Baldwin et al. 1997; Bell et al. 1993; Caress and Steinemann 2003; Meggs et al. 1996), with various kinds of elicited symptoms. Nevertheless, the pathophysiology behind the symptoms is unknown. A comprehensive term for chemical intolerance is “multiple chemical sensitivity” (MCS), which is associated with symptoms in more than one organ after the sufferer has been exposed to very low concentrations of chemical substances (Cullen 1987). Because no objective investigations have, until now, confirmed a diagnosis of MCS, the disorder has been rejected as an established organic disease (American Academy of Allergy 1999; Gots 1995; Staudenmayer 2001).

A subgroup of patients with airway complaints, reporting cough and other airway symptoms from scents and chemicals, had an increased capsaicin cough sensitivity (Millqvist et al. 1998; Ternesten-Hasseus et al. 2002b). This capsaicin-induced cough could be blocked by inhaled anesthesia, and it was suggested that sensory nerves are involved in the pathophysiology (Millqvist 2000). Furthermore, elevated levels of sensory nerve-mediated tachykinin substance P were found in nasal lavage fluid (NAL) of patients with chronic cough and increased capsaicin cough sensitivity (Cho et al. 2003). Consequently, we previously proposed a mechanism of increased sensitivity of the afferent nerves in patients with airway symptoms induced by scents and chemicals and suggested using the term “sensory hyperreactivity” (SHR; Millqvist et al. 1998).

Capsaicin, the ingredient that produces the heat in hot chiles, is a well-known cough-inducing agent when inhaled (Fuller et al. 1985; Karlsson et al. 1988; Midgren et al. 1992). Capsaicin stimulates unmyelinated, afferent sensory C-fibers. The capsaicin receptor, vanilloid receptor 1 (VR1), has been identified (Caterina et al. 1997). This receptor responds not only to capsaicin but may also participate in the detection of noxious thermal and chemical stimuli *in vivo* (Caterina et al. 1999; Tominaga and Julius 2000). In the skin, VR1 is essential to selective modalities of pain sensation and to tissue injury-induced thermal hyperalgesia (Caterina et al. 2000). There is also a link between pain and hyperalgesia, on the one hand, and neurotrophins and capsaicin, on the other (Anand 1995, 2004; Shu and Mendell 199b, 2001). Increased levels of serum nerve growth factor (NGF) have been reported in patients with MCS, and the levels rose 1.5-fold after provocation with irritating fumes (Kimata 2004).

Neurotrophins are a group of four similar proteins, which are essential for survival and axonal outgrowth and development of sensory neurons, including capsaicin-sensitive fibers (Chun and Patterson 1977; Levi-Montalcini 1987; Lewin and Barde 1996; Shu and Mendell 1999a, 2001). In the airways, the most important neurotrophin is believed to be NGF, but because it is derived primarily from nonneuronal sources, its role in patients with SHR is uncertain. Hypothesizing that a neurochemical alteration takes place in patients with SHR, we measured NGF levels in nasal secretions before and after capsaicin inhalation provocations and related the levels to capsaicin cough sensitivity.

## Materials and Methods

The study included 13 nonsmoking patients, 8 women and 5 men, 30–63 years of age (average age 50 years). They were referred to an asthma and allergy outpatient clinic because of symptoms suggestive of asthma or allergy, but, during the workup, this could not be verified. All participants had normal lung function [forced expiratory volume during 1 sec (FEV_1_) > 75% of predicted values], negative metacholine tests, and negative skin-prick tests (using a standard panel of 11 allergens). None demonstrated spirometric reversibility or variability in pulmonary function. Five patients regularly used β_2_-agonists and/or inhalation corticosteroids, but with no tangible effect. All patients had tried β_2_-agonists and inhalation corticosteroids, but 8 had stopped taking medication completely because of its lack of effect. None had a history of gastroesophageal reflux or heart disease.

The patients were screened using a questionnaire on airway symptoms and on symptoms in response to scents and chemicals. All had a history of at least 2 years of coughing and pronounced upper and lower airway symptoms induced by scents and chemicals. The patients had positive reactions to a capsaicin inhalation test, administered according to the method described by Johansson et al. (2002). They were diagnosed as having SHR as an explanation for their airway symptoms, in accordance with the guiding principles in this article, which recommend this diagnosis for patients with a combination of pronounced airway sensitivity to chemicals and increased capsaicin cough sensitivity.

The control group consisted of 14 healthy, nonsmoking subjects 29–69 years of age (average age 50 years), composed of 10 women and 4 men. None had a history of respiratory difficulties, asthma, or allergy (except for 1 person with an isolated positive reaction to birch), and none was on regular medication. Informed consent was obtained from all subjects at the start of the investigation, which was approved by the Hospital Ethics Committee in Gothenburg, Sweden.

We performed capsaicin inhalation provocation in accordance with previous studies (Millqvist et al. 1998; Ternesten-Hasseus et al. 2002b). One milliliter of saline was nebulized (Pariboy 36; Paulritzau Pari-Werk, Starnbergam-See, Germany) and inhaled to completion over 6 min to induce coughing, followed by 4 min rest. The patients did not use a nose clip during the provocations. We counted the number of coughs for 10 min from the onset of provocation and recorded them on tape. Thereafter, we carried out provocations in the same way, using increasing concentrations of capsaicin: 0.4, 2, and 10 μM in a 1-ml solution. We measured FEV_1_ with a spirometer (Vitalograph, Buckingham, UK) before and after each provocation and recorded the best of two trials. Following each provocation, we registered symptoms by scoring them on a scale of 0–3 (0 = no symptoms, and 1 = mild, 2 = moderate, and 3 = severe). Eleven main symptoms were analyzed: breathing problems, nasal irritation, throat irritation, hoarseness, chest pain/pressure, phlegm, eye irritation, dizziness, headache, fatigue, and sweating. The selection of symptoms was based on previous experience (Ternesten-Hasseus et al. 2002b).

### Collection of NAL and determination of nerve growth factor.

A 5-mL aliquot of room-tempered saline was instilled into the right nostril of the subjects before and directly after capsaicin provocation. The subject’s head was tilted back and after approximately 10 sec the head was raised and NAL expelled into a collecting basin, measured for volume, and then transferred into conical tubes. We measured the returned volume and centrifuged it at 400*g* for 10 min at 4°C. The supernatant was then frozen at −80°C. We measured concentrations of NGF in supernatant samples of pre- and postcapsaicin challenge NAL using an enzyme-linked immunosorbent assay (ELISA; Duo Set ELISA; R&D Systems, Abingdon, UK). Nerve growth factor in standard and samples was bound to an anti-NGF mouse monoclonal antibody coated onto a BD Falcon 96-well microplate (BD Biosciences, Bedford, MA, USA). After washing, we added biotinylated goat antihuman NGF and allowed it to bind to the NGF. After another wash, we added peroxidase-labeled streptavidin and allowed it to bind to the biotin on the goat antibodies. After a final wash, tetramethylbenzidine was added as an enzyme substrate. We added sulfuric acid as a stop solution and determined the amount of NGF by optical density using a microplate reader set at 450 nm (VMax microplate reader; Molecular Devices, Sunnyvale, CA, USA). We used recombinant human NGF as standard, ranging from 15.6 to 2,000 ng/L. The assay had a lower detection limit of 2 pg/mL. To test the specificity of the assay, we performed double ELISA measurements of NGF on each specimen.

### Statistics.

We analyzed the results obtained with different doses in the two groups using the Mann-Whitney *U*-test for nonpaired data and the Wilcoxon signed-rank test for paired data. Data are presented as means and 95% confidence intervals (CIs), and a *p-*value of < 0.05 was taken as statistically significant.

## Results

### Outcome of the capsaicin provocations.

Both patient and control groups demonstrated a dose-dependent response to capsaicin inhalation. In the patient group, the mean number of coughs was 21 (95% CI, 0–44), 58 (95% CI, 34–73) and 86 (95% CI, 74–98) for the three doses, respectively. The corresponding values for the control group were 6 (95% CI, 0–13), 17 (95% CI, 12–23), and 37 (95% CI, 26–48). With each dose of capsaicin, the patient group coughed significantly more than the control group (*p* < 0.01 for the 0.4 μM dose and *p* < 0.001 for both 2 and 10 μM doses).

In addition to increased coughing in patients, capsaicin provocations induced other symptoms, the most common being throat irritation, heavy breathing, eye irritation, phlegm, and rhinorrohea. The control subjects had no, or only a few, symptoms induced by capsaicin inhalation. The mean FEV_1_ in the patient group was 99% (95% CI, 90–108) of that predicted before capsaicin inhalation provocations, and no significant differences were found after the provocations.

### Nerve growth factor in NAL.

**Figure 1:**
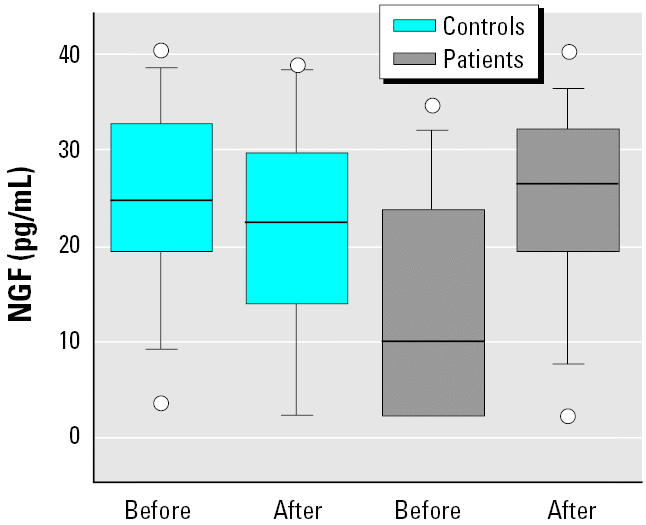
Box plot of NGF levels in 13 patients with airway symptoms induced by scents and chemicals and 14 control subjects before and after inhalation provocation with three concentrations of capsaicin. See “Materials and Methods” for details. The horizontal line in the center of each box is the median. The top and bottom of the box represent the 25th and 75th percentiles, and whiskers indicate the 10th and 90th percentiles. Circles are individual maximum and minimum data points.

The double ELISA measurements of NGF, performed on each specimen, were constant, without significant differences in the outcome of the results from capsaicin provocations. Basal levels of NGF were significantly lower (*p* < 0.02) in the patient group: their mean value was 13.8 pg/mL (95% CI, 6.3–21.4) compared with control subjects, who had mean values of 25.1 pg/mL (95% CI, 18.9–31.2). After capsaicin inhalation, there was an increase in NGF in the patient group (mean increase after provocation: 11.1 pg/mL; 95% CI, 3.1–19.1; *p* < 0.01) but not among control subjects, who displayed a decrease in NGF (mean decrease after provocation: 3.2 pg/mL; 95% CI, −1.6 to 8.1). The change in NGF levels after capsaicin provocation differed significantly between the two groups (*p* < 0.005; Figure 1).

**Figure 2:**
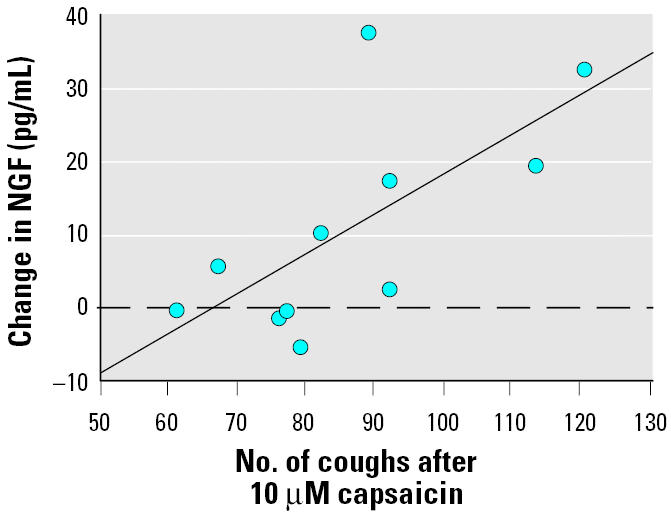
Correlation between change in NGF after provocation with three concentrations of capsaicin and number of coughs after inhalation of the highest dose of capsaicin (10 μM). *r* = 0.7.

In the patient group, there was a statistically significant correlation between the number of coughs after the highest inhaled capsaicin dose (10 μM) and the change in NGF levels in NAL after provocation (*p* < 0.01, *r* = 0.7; Figure 2). After the highest inhaled capsaicin concentration, each of the symptom scores for throat irritation, phlegm, and rhinorrohea correlated significantly (*p* < 0.004, *r* = 0.8; *p* < 0.04, *r* = 0.6; and *p* < 0.04, *r* = 0.8, respectively) with the change in NGF levels in NAL after provocation.

## Discussion

The main finding of this study is that patients with SHR do have enhanced cough sensitivity to inhaled capsaicin, which correlates to a small but significant increase in NGF in NAL after capsaicin provocation. This indicates a neurochemical imbalance of the respiratory system in patients with SHR. Because there are similarities in symptoms and capsaicin sensitivity between patients with SHR and patients with MCS (Ternesten-Hasseus et al. 2002a), the groups probably overlap each other.

Compared with patients with asthma and allergy, the levels and increase of NGF are discrete and emphasize the discrepancy between these two conditions, though these groups have similar airway symptoms and are often confused. Recent studies have shown an interplay between NGF and airway inflammation. Increased levels of NGF have been found in the serum, bronchial tissue, and bronchoalveolar fluid of patients with allergy and asthma (Bonini et al. 1996; Noga et al. 2001, 2003; Olgart Hoglund et al. 2002). In patients with allergic rhinitis, levels of NGF in NAL were increased after allergen provocation compared with control subjects (Sanico et al. 2000).

Among the patients in our study, the lower basal NGF levels in NAL may seem surprising, but this finding underlines that patients with SHR do not have mucosal inflammation, which is probably the main source of high airway NGF levels in asthma and allergy sufferers. However, in this study, the NGF reaction induced by capsaicin was evident among patients and may be derived from hyperreactive nerve endings. The nervous system of patients with SHR may have an ability to overrespond to noxious stimuli with NGF production, which, in the long run, is followed by depletion of basal neurotrophin levels. Because the original source of NGF is unknown, one possibility is that the measured levels of NGF reflect plasma levels or levels in the lower airways. In control subjects, levels of NGF were unchanged, with a tendency toward lower values after capsaicin provocation. This indicates a stable system in the control group, where, after two nasal lavages, some of the NGF had been rinsed out.

After capsaicin challenge, the symptom scores for rhinitis had a strong, but unsurprising, correlation with an increase in NGF, because the nasal mucosa may have produced the factor being analyzed. However, the mechanisms behind the reaction are unclear: it could be reflex mediated; a small amount of capsaicin could have reached the nose from the pharynx; or some of the nebulized solution could have been dispersed and inhaled through the nose because the patients did not use a nose clip.

The NAL technique involves dilution of the expelled nasal secretions. We did not estimate the dilution factor, which is a possible source of error in the measured NGF. Most patients complained of rhinorrohea after capsaicin provocation, which may have contributed to a further dilution of NAL in patient group compared to controls. However, after provocation, there was an increase in NGF per milliliter of NAL and also a correlation with a high rhinorrohea score in the patient group; therefore, the dilution effect should not invalidate the results. Because of the dilution of NAL, the absolute NGF values must be regarded as uncertain and conclusions could be drawn only from group levels. In future research, albumin levels should also be measured to compensate for the dilutional effect. Another source of error for measured NGF may have been the influence of medication in the patient group. Five of the patients were on regular medication with β_2_ agonists or inhaled steroids, both of which are known to suppress NGF production (Amann and Schuligoi 2004; Noga et al. 2001).

Previously, an abnormality of the sensory nervous system in the airways was suggested in the pathophysiology of patients with symptoms induced by chemicals (Meggs 1995). Recently, Kimata (2004) recorded increased plasma levels of substance P, vasoactive intestinal peptide, and NGF in patients with self-reported MCS, which support this theory. This theory is also corroborated by the findings of augmented cough sensitivity to inhaled capsaicin (Johansson et al. 2002; Millqvist 2000; Millqvist et al. 1998, 2000; Ternesten-Hasseus et al. 2002b). Increased levels of substance P were found in NAL of patients with chronic cough but without bronchial hyper-responsiveness; there was also increased capsaicin cough sensitivity compared with controls (Cho et al. 2003). The conditions of chronic cough and airway sensitivity to scents and chemicals are closely related. Many patients with SHR complain of daily problems with cough (Millqvist et al. 1998, 2000; Ternesten-Hasseus et al. 2002b). However, it is not known how often patients with a diagnosis of chronic cough are also intolerant to scents and chemicals. In both conditions, there is no well-defined explanation, but neurochemical findings allude to analogous mechanisms.

In animals, NGF acutely conditions the response to capsaicin, suggesting that NGF may be important in sensitizing the response of sensory neurons and may play a role in pain and hyperalgesia (Anand 1995, 2004; Shu and Mendell 1999b, 2001). Initial interpretation of the findings in the present study suggests that an alteration of neurochemical balance in the airways is related to SHR, resulting in cough and other airway symptoms. We hypothesize that a type of mucosal hyperalgesia has occurred in patients with SHR. The upper and lower airways could mirror each other by trigeminal and vagal reflexes—the two main sensory nerves of the airways. Neuropeptides released from the lower airways may also induce symptoms in the other end of the airway system and vice versa. The term “united airways” is often used with reference to asthma and allergy, but it is probably, more or less, suited to most conditions in the airways. A sensory hyperreactivity after contact with chemical stimuli, in concentrations normally regarded as harmless, may result in symptoms mimicking those usually elicited by noxious stimuli and resulting in symptoms in both the upper and the lower airways. Other neurochemical factors, such as neuropeptides, will need to be analyzed in the future.
